# Negative allosteric modulation of mGlu7 disrupts fear memory reconsolidation and glutamatergic signaling in rat and human brain tissue

**DOI:** 10.1038/s41380-025-03202-x

**Published:** 2025-12-23

**Authors:** Alexandru Cristian Ciobanu, David Mota Caseiro, Ruifang Niu, Rodrigo Triana del Rio, Cédric Leroux, Alessio Stefanelli, Carmen Flores Nakandakare, Etienne Pralong, Roy T. Daniel, Robert Lütjens, Erwin H. van den Burg, Ron Stoop

**Affiliations:** 1 Center for Psychiatric Neurosciences, Lausanne University Hospital Center (CHUV), Prilly-Lausanne, Switzerland; 2 Section of Neurosurgery, Department of Clinical Neurosciences, Lausanne University Hospital Center (CHUV), Lausanne, Switzerland; 3 Neurosterix, Chemin des Mines 9, Campus Biotech, CH-1202 Geneva, Switzerland

**Keywords:** Neuroscience, Psychiatric disorders

## Abstract

Anxiety- and stress-related disorders are amongst the most frequent neurological disorders, and efficient treatment is lacking. Metabotropic glutamate receptors (mGlu) have emerged as promising targets for intervention. Of particular interest is mGlu7, because of its expression in the lateral amygdala (LA), a region critical for fear learning. In the present study we examined the effects of the highly specific negative allosteric modulator of mGlu7 (ADX71743) on fear memory reconsolidation. Our investigation unveils that infusion in rats of ADX71743 in the LA or subcutaneously disrupts the reconsolidation of fear memories. This effect on reconsolidation was specific to the conditioned stimulus (CS), required fear memory recall, occurred in a defined time window after recall, and significantly decreased reinstatement of fear. Moreover, in ex vivo experiments, ADX71743 disinhibited glutamate release, as evidenced by increased spontaneous excitatory postsynaptic currents (EPSCs) frequency and enhanced amplitude of electrically and optogenetically evoked EPSCs at thalamus-to-LA synapses. Conversely, under high-stimulation conditions, ADX71743 attenuated transmission as demonstrated by the complete prevention of long-term potentiation (LTP) at thalamus-to-LA synapses. Finally, application of ADX71743 to human brain tissue mirrored the increased frequency of spontaneous EPSCs observed in the rat LA, underscoring translational relevance. Our findings highlight negative allosteric modulation of mGlu7 as a novel therapeutic avenue for addressing anxiety- and fear-related pathologies, bolstered by the congruent effects of ADX71743 on glutamatergic transmission across species.

## Introduction

Anxiety, fear and stress-related disorders represent major groups of neurobiological disorders, affecting over 15.7 million people each year in the US alone [1]. Globally, one in three people will grapple with these disorders at least once in their lifetime [2]. Unfortunately, current behavioral and pharmacological treatments suffer from a high rate of relapse, and achieve only partial remission of symptoms, highlighting the need for improved or new treatment options [3].

Since their discovery, metabotropic glutamate receptors (mGlus) have been proposed as novel targets for anxiety-related disorders [4]. The family of mGlus consists of eight members, mGlu1 – mGlu8, categorized into three groups based on their signaling pathways and synaptic localization [5]. Despite promising early preclinical and clinical results, neither targeting group I family member mGlu5 with the antagonist MPEP in rodents [6] or the negative allosteric modulator fenobam [7], nor targeting group II family members mGlu2/3 with the agonist LY354740 [8] has translated so far into an FDA-approved treatment of anxiety- and fear-related disorders in human patients.

Other preclinical investigations have focused on mGlu7, a member of Group III, to study its potential as an anxiolytic target. Reduced anxiety can be observed in mGlu7 KO mice, and in wild types after downregulating mGlu7 expression by si-RNA [9–11] or after its inhibition by an orthosteric-like antagonist, XAP044 [12]. Additionally, the subcutaneous administration of ADX71743, a novel compound targeting mGlu7 specifically through negative allosteric modulation, exhibited anxiolytic effects across a battery of tests in both mice and rats [13]. These findings highlight the promising potential of mGlu7 negative allosteric modulation for treatment of anxiety- and fear-related disorders.

mGlu7 is widely expressed mostly presynaptically throughout the brain, including in regions such as the thalamus and the lateral amygdala (LA), where it may exert its anxiolytic action [14, 15]. Synaptic plasticity in thalamic projections onto pyramidal neurons in the LA is critical for fear learning and fear memory storage [16, 17], with aberrant glutamate signaling at these synapses implicated in posttraumatic stress disorder (PTSD) [18]. mGlu7 signaling has mostly been studied in the cortex and found to modulate glutamate release bidirectionally: under baseline or low frequency stimulation, presynaptic mGlu7 is constitutively active and suppresses glutamate release by autoinhibition [19] via presynaptic inhibitory G protein α_i/o_ subunits and interactions with postsynaptic proteins Elfn1 and Elfn2 [20–23]. Conversely, during high frequency stimulation, the mGlu7-Elfn1 complex promotes glutamate release by engaging presynaptic Ca^2+^-permeable kainate receptors, and presynaptic Munc-18 [24], to generate strongly facilitating synapses [21].

Interestingly, it has been discovered fear memories are not permanently fixed, but can weaken following a memory recall and then strengthened again through “reconsolidation”. This process allows for the integration of new experiences into the original memory trace [25]. Disrupting reconsolidation presents a potential therapeutic approach for anxiety- and fear-related disorders, as it could weaken, or perhaps even erase, the fear memory trace. Reconsolidation thereby contrasts with fear extinction, a process that seems to involve an active inhibition of the associative memory between conditioned (CS) and unconditioned stimulus (US), that leaves the original fear trace unaltered [26, 27]. The original memory can thus still return, as a result of “spontaneous recovery” or, more actively, by “reinstatement of fear”, which causes a relapse of previous fear responses to the CS following re-exposure to the US alone [28–31].

A first successful pharmacological interference with memory reconsolidation was shown with the β-adrenergic receptor (βAR) antagonist propranolol, which, when applied after fear memory recall, disrupts fear memory reconsolidation [32–34]. Propanolol has started to be used in combination with psychotherapy for the treatment of PTSD [35] and phobias [36]. However, its effects are not consistently found in animals or in humans [37–41], and there is currently some debate about the best timing of propranolol application, i.e. immediately before or after fear memory recall [42, 43]. Moreover, the precise mechanisms by which propranolol acts are not precisely clear: whether it only disrupts reconsolidation, also enhances extinction, or simply prevents access to the fear memory and expression of fear. Additionally there is debate about whether propranolol selectively removes only the emotional component of the fear memory [44, 45].

Considering the potential of fear memory reconsolidation and the inconsistent results with propranolol in preclinical and clinical studies, we addressed the question of whether mGlu7 could constitute a novel target for disruption of fear memory reconsolidation, based on its bidirectional modulation of synaptic strength and its involvement in fear memory. We hypothesized that the recently developed, highly selective negative allosteric modulator of mGlu7 ADX71743 could interfere with fear memory reconsolidation in the LA of rats. Our results demonstrate that ADX71743 disrupts fear memory reconsolidation following direct injection into the LA as well as after peripheral administration. Additionally, electrophysiological studies revealed that ADX71743 in thalamic-LA projections acutely enhances basic glutamatergic synaptic transmission while blocking the induction of long-term potentiation (LTP) through a presynaptic mechanism of action. In addition, similar stimulatory effects of ADX71743 on glutamatergic synaptic transmission were observed in vitro in amygdala and cortical tissue from human subjects. These findings bring to light the potential of mGlu7 negative allosteric modulators as promising candidates for pharmacological disruption of fear memory reconsolidation, which may ultimately translate into treatments in human subjects for PTSD and phobia.

## Materials and methods

### Animal subjects

Male Sprague Dawley rats, aged 4 to 8 weeks (to match electrophysiological and behavioral studies) were group-housed under a 12 h light/dark cycle with food and water ad libitum, and single-housed after cannula implantation for 1 week before the experiments started. All procedures were approved by the veterinary office of the Canton de Vaud, Switzerland under license VD3565X.

### Human brain tissue

Human brain tissue was obtained from four temporal lobe epileptic patients who were resistant to pharmacological treatment and therefore underwent surgery to remove the epileptic focus. Patients ranged in age from 9 months to 54 years and had been diagnosed with intractable epilepsy based on the Phase I evaluation performed at the Geneva pre-surgical epilepsy unit and discussed at the Lausanne-Geneva Epilepsy Surgery Board. Dissected brain tissue was collected in ice-cold oxygenated artificial cerebrospinal fluid (ACSF) and prepared for patch clamp electrophysiology. Patients or their parents gave written consent and tissue collection was approved by the ethical committee of the Canton de Vaud, Switzerland (license 449/14).

### Cannula implantations and virus injections

All surgical stereotaxic procedures were performed under isoflurane anesthesia and semi-sterile conditions in eight weeks (for subcutaneous application of ADX71743), seven-weeks (for cannula implantations) or four weeks (for virus injections) old male rats. Before surgery, lidocaine (Rapidocain®; 10 mg/ml, Sintetica, Switzerland) was administered subcutaneously at the incision site. Following surgery, rats received an antibiotic locally. Body temperature was maintained with a heating pad. Animals were allowed to recover for one week prior to testing and were handled daily by the experimenter. For virus injections, animals were sacrificed four weeks after surgery to allow optimal virus expression.

For analysis of local effects of ADX71743 on fear memory reconsolidation, indwelling bilateral guide cannulas (stainless steel, 23 G, 12 mm long) were implanted 2 mm above both the left and right LA (AP: −2.93 mm relative to bregma, ML: ±5.33 mm lateral; DV: −6.4 mm below the surface of the skull [46]), and anchored to the skull using dental acrylic cement. Injectors targeted the dorsal part of the LA, 1 mm below the tip of the guide cannulas. The guide cannulas were kept viable with dummies, which were removed daily and cleaned during handling.

rAAV/CamKIIa-hChR2(H134R)-mCherry virus (UNC Vector Core, USA; titer: 3 × 10^12^ vg/ml) was injected (1 μl, 0.15 μl/min) in the medial part of the thalamic medial geniculate nucleus (MGM; antero-posterior: −5.64; medio-lateral: ± 3.00; dorso-ventral: −6.00 mm relative to Bregma [46]), important for auditory fear conditioning [47], and projecting to the LA [48].

### Fear conditioning

Fear conditioning was performed with a computerized fear conditioning system (Med Associate Inc., St. Albans, Florida, USA). The conditioning chamber consisted of a transparent Perspex box enclosed in a wooden chamber to reduce external noise and visual stimulation. The floor consisted of a removable stainless-steel grid connected to a computer-controlled shock delivery unit for the automatic delivery of foot shocks, programmed in med-PC (Med Associate Inc., St. Albans, Florida, USA). A video camera at the top of the chamber enabled video recording.

Rats were placed in the conditioning chamber (35 ×22 x 40 cm) with cinnamon scent on day 1, and allowed to habituate for 5 min. Then they received 3 pairings of an auditory conditioned stimulus (CS; 30 s, 5 kHz, 80 dB) with an electric foot shock (unconditioned stimulus (US); 2 s, 0.5 mA co-terminating with the CS). Pairings were given at random intervals (60 – 120 s). Fear memory recall on day 2 consisted of one CS presentation [32] in a different chamber (45 × 22 × 40 cm) with a smooth plastic floor and a lemon scent. Fear memory reconsolidation was assessed on day 5 with 5 CS presentations in a retention test in a third context, which was a hexagonal cage of carton boards 29 cm height and 40 cm diagonal. To assess fear reinstatement, animals were exposed to the US once on day 11 to reactivate the fear memory trace [30], and to one CS presentation on day 12.

To determine the effects of ADX71743 in the LA on fear memory reconsolidation, control male rats (8 weeks of age) received ACSF + DMSO (0.01% final concentration; vehicle controls), or 1 µM of ADX71743 in vehicle through cannulas implanted in the LA (0.4 µl, 0.5 µl/min) 10 min or 4 h after the recall of the CS. To determine the specificity of ADX71743 for the CS, animals were alternately fear-conditioned on day 1 to a 5 kHz and 15 kHz tone. Previously we had found that these tones are sufficiently diverse to prevent overlying effects in a social buffering of fear memory protocol [49]. One of the two tones was recalled for any particular rat on day 2. Those recalled for the 5 kHz tone received ADX71743, and those recalled for the 15 kHz tone received vehicle. On day 3, tone recall, in a different context (EU 3H rat cage, top 42.5 × 26.5 cm, bottom 37.5 × 21.5 cm, height 18 cm) and injections were reversed for each rat so that for each rat ADX71743 was paired with the 5 kHz tone only. The injectors were left in position for 2 additional minutes after drug infusion ended. On day 5 in the retention test, each CS was presented five times consecutively, with some rats receiving the 5 kHz tone first, and others receiving the 15 kHz tone first.

To determine the effects of peripherally administered ADX71743 on fear memory reconsolidation, vehicle or ADX71743 was infused subcutaneously within 10 min after fear recall on day 2 (100 mg/kg bodyweight, 1 ml). ADX71743 was dissolved in 50% (2-hydroxypropyl)-β-cyclodextrin (Sigma Aldrich; [13]) and propranolol in saline for intraperitoneal administration (10 mg/kg bodyweight, 1 ml [32]).

Freezing behavior, the defensive response of rats with cessation of all movement except breathing, was taken as a measure of fear, and analyzed off-line by two experienced researchers blinded to the treatment, or by automated analysis using Ethovision (Noldus), using 2 seconds as threshold for freezing. Presented freezing values reflect average freezing behavior during 5 min of habituation or during and 30 seconds after the CS.

### In vitro whole cell patch clamp electrophysiology

Patch clamp electrophysiology was performed as previously described [50–52]. Rat and human brain tissues were sliced and processed similarly. Principal cells were identified on the basis of spike frequency adaptation following depolarizing current injection, the presence of an Ih current [53, 54] and their morphology [55, 56]. Intrapipette solutions were based on K-methylsulfate (135 mM) or for LTP experiments on K-gluconate (135 mM).

eEPSCs were induced by electrical stimulation of the internal capsule, containing descending thalamic fibers to the LA. The bipolar electrode was positioned medially to the LA and dorsally to the central amygdala in the ventral striatum. Stimuli of 100 µs were delivered with an Iso-Flex stimulus isolator (AMPI, Jerusalem, Israel) in pairs with an interpulse interval of 40 ms. For optogenetic stimulation of thalamic axon terminals in the LA, Channelrhodopsin 2 was excited with blue light (473 nm) during 5 ms with Luxeon emitter LXHL-LB3C (Luxeon III Star power light source, Lumileds, USA), with radiometric power of 13.9 mW.

The LTP induction protocol was taken from Weisskopf et al. [57], with 100 µM of picrotoxin in the bath to block GABAergic signaling.

For each electrophysiological experiment two to five slices per rat were used, and one cell per slice was recorded. ADX71743 (Addex Therapeutics, Geneva, Switzerland) was dissolved in DMSO, then freshly diluted in ACSF (final DMSO concentration was 0.005%) and bath-applied in ACSF to a final concentration of 500 nM.

### Statistical analyses

Animals were assigned to a group by using an online random number generator (https://www.random.org/). Group sizes were estimated on the basis of data and approximate effect size on the effects of anisomycin injections in the LA [25], which was 6 animals per group. As we expected ADX71743 to have milder modulatory effects than those of a protein synthesis blocker, we aimed for at least 9 animals per group in our initial experiments. Rats freezing less than 20% during fear recall were excluded, as no fear memory had formed and reconsolidation could not be tested. Animals with cannulas outside the LA were excluded from the analysis, but are shown to indicate the specificity of effects in the LA. Signs of inflammation at the injection site was a criterion for exclusion; this applied to one rat that had received a subcutaneous injection. Each experiment has been replicated by three (electrophysiology) or four (behavior) experienced scientists. Statistical differences between freezing behavior of vehicle- and ADX71743-injected rats across sessions were assessed by a three-way or two-way ANOVA, followed by Bonferroni corrections using Origin (OriginLab Corporation, Northhampton, MA, USA), GraphPad Prism (GraphPad Software, San Diego, California USA), and SPSS (IBM Corp, Armonk, NY, USA). Normality of the data was confirmed with the Shapiro-Wilk test. Data analysis of electrophysiological recordings was performed using pClamp 10.2 (Axon Instruments, Foster City, CA) and Minianalysis (Synaptosoft Inc., NJ, USA). Spontaneous currents were assessed using Minianalysis software (Synaptosoft Inc., Chapel Hill, NC, USA) with the threshold for detection set two to three standard deviations above the baseline noise. eEPSC amplitudes were normalized to the average of baseline amplitudes. Effects of ADX71743 were assessed by comparing EPCS frequencies and amplitudes during 6 min of stable baseline recording and during drug application. Statistical significance was assessed, after confirmation of normal distribution with the Shapiro-Wilk test, by using the Student’s *t* test, paired for comparisons between the predrug (baseline) and drug exposure conditions (so that each cell served as its own control), or unpaired for comparisons between different cell groups (as in the LTP study), using Origin. Sample size, number of replicates, and statistical tests are specified in each figure legend. Data are expressed as means ± SEM.

## Results

### Negative allosteric modulation of mGlu7 in the LA disrupts fear memory reconsolidation

**Fig. 1:**
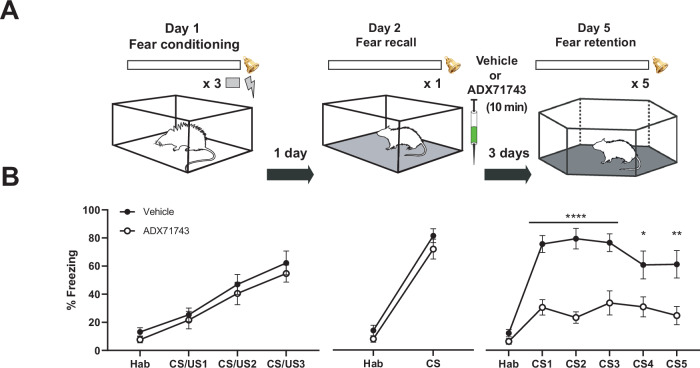
ADX71743 disrupts fear memory reconsolidation in the LA. **A** Experimental protocol. Rats were fear-conditioned by three tone (30 s, 5 kHz, 80 dB) – foot shock (0.5 mA) pairings on Day 1 (CS + US). Immediately after fear recall by presenting the tone once on Day2 (CS), ADX71743 (1 µM) or vehicle solution (ACSF containing 0.01% DMSO) was infused into the LA. Fear memory retention was tested on Day 5 by presenting the tone five times (CS 1–5). Three different contexts were used for the different sessions of the experimental protocol. **B** ADX71743 infused in the LA bilaterally immediately following fear retrieval suppressed freezing during the fear memory retention test three days later. Two-way ANOVA with repeated measures, F(1,19) = 30.9, *p* < 0.0001; *p = 0.0125, **p = 0.0012, p****<0.0001, Bonferroni corrected, *n*(vehicle)=10, *n*(ADX71743) = 11.

To assess whether ADX71743 in the lateral amygdala (LA) can affect fear memory reconsolidation, we fear-conditioned male rats on day 1 to a 5 kHz tone (the conditioned stimulus, CS), reactivated fear memory 24 h later by a single CS, and infused the compound or vehicle solution through cannulas in the LA 10 min later. Fear memory reconsolidation was assessed three days later, on day 5, in a fear memory retention test [32] (Fig. 1A). Fear conditioning on day 1, and fear memory recall on day 2 induced similar levels of freezing in both groups (Fig. 1B). During the fear memory retention test on day 5, rats that had been infused with ADX71743 showed significantly reduced freezing levels across the five consecutive CS presentations compared to rats that had received vehicle control (Fig. 1B). This result is a first indication that application of the negative allosteric modulator of mGlu7 had indeed disrupted fear memory reconsolidation. Postmortem analysis of the canula locations (Supplementary Fig. S1A, grey squares and triangles), revealed two rats in which ADX71743 had been infused outside the LA. These rats failed to show reduced freezing in the fear memory retention test (Supplementary Fig. S1B), confirming that the disruption of memory reconsolidation by ADX7143 was specific to the LA.

To further test whether ADX71743 selectively disrupted fear memory reconsolidation rather than impairing general fear recall, we used a two-tone paradigm with two distinct CSs, a 5 and a 15 kHz tone [49]. In this paradigm, rats received ADX71743 10 min after exposure to CS1 and vehicle 10 min after CS2 in the fear memory recall session. To prevent residual drug effects on the same day, these pairings were conducted interchangeably on consecutive days. Half of the animals received on day 2 ADX71743 after CS1 and on day 3 vehicle after CS2. The other half received on day 2 vehicle after CS2 and on day 3 ADX71743 after CS1. During the retention test, on day 5, all rats showed significant reduced freezing to CS1 (paired with ADX71743) compared to CS2 (paired with vehicle) (Supplementary Fig. S2). These findings demonstrate that the effects of ADX71743 are selective for the tone that has been recalled and do not result from a general fear recall impairment.

Earlier experiments with the protein synthesis blocker anisomycin [25] have shown that reconsolidation cannot be disrupted beyond six hours after fear recall. To further define the critical time window for ADX71743 effectiveness in reconsolidation, we conducted additional experiments in which the compound was administered four hours after CS exposure instead of 10 min. This delayed administration still resulted in a significant reduction in freezing on day 5 compared to vehicle injections, suggesting that reconsolidation is not complete at this later time point (Supplementary Fig. S3). On the other hand, ADX71743 infused 24 h after CS exposure had no effect any more on fear memory recall on day 3 or day 5 (Supplementary Fig. S2). These findings indicate that the critical time window for administration of ADX71743 closes somewhere between 4 and 24 h after CS exposure, which is in line with the six hours limit for reconsolidation identified earlier [25].

### Disruption of reconsolidation by peripheral ADX71743 requires recall and reduces reinstatement

**Fig. 2:**
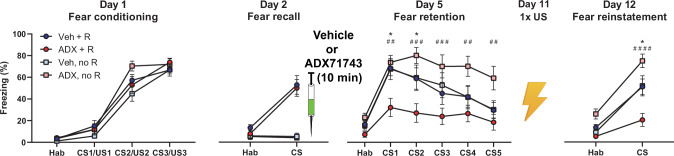
The need for fear memory recall and the weak reinstatement of fear indicate disruption of fear memory reconsolidation following subcutaneous injection of ADX71743. Male rats were divided over four groups based on treatment and the presence or absence of fear recall (Veh + R: vehicle plus recall; ADX + R: ADX71743 plus recall; Veh, no R: vehicle without recall; ADX, no R: ADX71743 without recall). All animals learned the association between the tone and the mild electric foot shock on Day 1. On Day 2, fear was recalled in half of the animals, which was followed by vehicle (50% (2-hydroxypropyl)-β-cyclodextrin) or ADX71743 (100 mg/kg bodyweight). The fear retention test on Day 5 revealed a strong reduction of fear in animals that had received ADX71743, but only if fear memory had been recalled on Day 2. No statistically three-way interaction in the retention test (F(5, 252) = 0.486, *p* = 0.786, using treatment (veh, ADX), recall session (yes, no) and trials (CS presentations) as factors, but with interaction between treatment and recall in a subsequent two-way ANOVA (F(1, 272) = 20.679, *p* < 0.001), indicating a specific effect on reconsolidation rather than a general effect on freezing behavior. p = 0.0004; *p < 0.05 ADX71743+recall vs other groups, ##*p* < 0.01, *p*###<0.001 ADX71743 without recall vs other groups, *n*(vehicle+recall)=13, *n*(ADX71743+recall)=13, *n*(vehicle-recall)=11, *n*(ADX71743-recall)=14. Following reactivatio*n* of fear memory on Day 11 by a single foot shock, the reinstatement of fear was weak in ADX71743-treated animals in which fear memory had been recalled on Day 2. Absence of significant three-way interaction (F(1, 84) = 1.439, *p* = 0.234), and with interaction between treatment and recall (F(1,88) = 9.505, *p* = 0.003), again indicating specific effects of ADX71743 on reconsolidation *p < 0.05 ADX71743+recall vs vehicle groups, ####*p* < 0.0001 ADX71743+recall vs ADX71743-recall. All tests Bonferroni corrected. CS induced limited freezing behavior in ADX71743+recall animals. Mann-Whitney test, Habituation vs CS, U = 37.5, *p = 0.015 (orange).

Disruption of fear memory reconsolidation implies that a fear memory recall is necessary on day 2 to reduce freezing behavior in the retention test on day 5. To confirm this for ADX71743, we administered the compound with or without fear memory recall. For these experiments, instead of a direct administration through cannulas in the LA, we injected ADX71743 subcutaneously (at 100 mg/kg bodyweight) to simultaneously test for its peripheral efficacy. Previous pharmacokinetic analyses showed that, following subcutaneous injections, ADX71743 concentration attains a maximum of approximately 3.3 µM 0.7 h after injection (as compared to 1 µM after cannula administration) and decays with a t_1/2_ of 90 min [13]. This maximum concentration represents approximately 30 times the in-vitro IC50, so we can reasonably assume a full blockade of mGlu7 at the time of memory reconsolidation [13, 25]. Subcutaneous application of ADX71743 after fear memory recall reduced freezing behavior in the fear memory retention test three days later to similar extent as previously found following local infusion in the LA (compare Fig. 2 with 1B). This was comparable to an intraperitoneal injection of propranolol (Supplementary Fig. S4), which has been shown to disrupt fear memory reconsolidation in humans and rodents [32, 35, 36]. Furthermore, and importantly, when the recall was omitted, injection of ADX71743 no longer diminished freezing in the retention test on day 5. This latter finding demonstrates that the recall (and inherent memory weakening) is indeed necessary for the ADX71743-induced reduction of freezing in the fear retention test, and provides further proof that ADX71743 interferes with fear memory reconsolidation (Fig. 2).

If ADX71743 indeed permanently weakens, or perhaps even erases, the fear memory trace, then we also expect that reinstatement of fear will not or hardly occur. We tested this hypothesis by extending the protocol to include a reinstatement paradigm in which the rats received one US (without the CS) on day 11 to reactivate the fear memory [30], and the CS on day 12 to test for reinstatement of the fear memory. We found that rats that had been injected with vehicle, or in which fear memory recall had been omitted, on day 2 froze substantially to the CS. In contrast, the rats that had received ADX71743 after fear memory recall on day 2 froze significantly less than the animals from the three other groups, suggesting that the fear memory trace had weakened but was not erased (as freezing to the CS was higher than during habituation, Habituation 5.2% vs CS 20.4%, Fig. 2). Taken together, these results indicate that negative allosteric modulation of mGlu7 can disrupt fear memory reconsolidation.

### ADX71743 promotes spontaneous presynaptic glutamate release in the rat LA

**Fig. 3:**
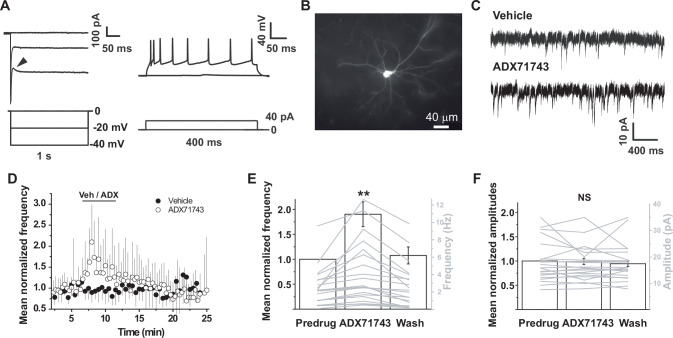
Bath-application of ADX71743 increases EPSP frequency, but not amplitude, in LA pyramidal neurons. **A** Ih current (arrowhead) following 20 and 40 mV hyperpolarizations (left panel) and action potential frequency accommodation following injection of 40 pA current (right panel). **B** Biocytin staining after patch-clamping showing a pyramidal neuron with distinct triangular shape. **C** Sample example traces showing spontaneous EPSCs before (top), and during (bottom) ADX71743 application (500 nM) under TTX (1 µM). **D** Transient effect of bath application of ADX71743 (500 nM) on spontaneous EPSC frequency (*n* = 21); vehicle control (0.005% DMSO in ACSF) is without effect (*n* = 6). **E** Quantification of mean effect of ADX71743 (500 nM) on normalized EPSC peak value of ADX71743-sensitive cells (one-tailed paired Student’s *t* test, predrug vs ADX71743, t = 3.57, **p = 0.0019, *n* = 21). Grey lines, frequency changes of individual cells (right axis). **F** Absence of effect of ADX71743 (500 nM) on spontaneous EPSC amplitude, taken at EPSC frequency peak of each responding cell (one-tailed paired Student’s *t* test, t = 0.91, ns *p* > 0.05, *n* = 21).

To determine, in vitro, the effects of ADX71743 on glutamatergic signaling, we first assessed its effects on spontaneous, low frequency glutamatergic synaptic transmission. We bath-applied ADX71743 (500 nM, 6 min) in a patch clamp electrophysiology setup and recorded spontaneous excitatory postsynaptic synaptic currents (sEPSC) from principal neurons (in the presence of TTX to block action potentials). Recorded principal neurons (see Methods) were regularly spiking cells or showed different degrees of action potential frequency accommodation following current injections (Fig. 3A). Biocytin staining revealed a distinct triangular shape of their soma [53, 54] (Fig. 3B). Recorded cells had an average resting membrane potential of −68 ± 2 mV, membrane resistance of 236 ± 36 MΩ, and membrane capacitance of 98 ± 10 pF.

In these neurons, ADX71743 transiently increased sEPSC frequency, peaking two min after the compound had reached the bath, and reverting to predrug frequency by the end of the incubation period (Fig. 3C–E). sEPSC amplitudes were not influenced by ADX71743 application (Fig. 3F). The increased sEPSC frequency and the lack of effect on sEPSC amplitude suggest that ADX71743 inhibits mGlu7 presynaptically, consistent with the reported presynaptic localization of the receptor [15] and enhanced constitutive or tonic [21, 58] activity of mGlu7 in the presence of ADX71743.

### ADX71743 enhances evoked synaptic transmission at thalamus-to-LA synapses

**Fig. 4:**
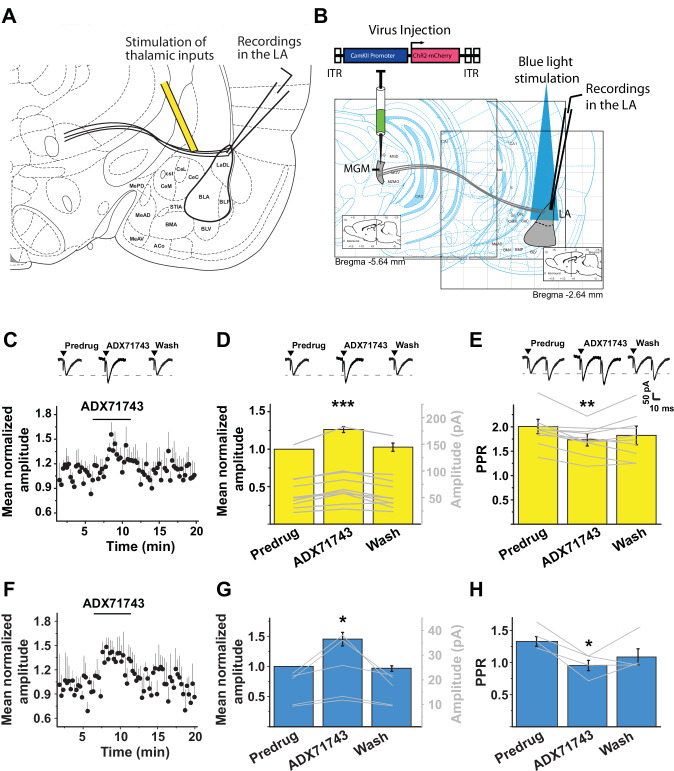
ADX71743 increases electrically and optogenetically evoked EPSC amplitude at thalamus-to-LA pyramidal neuron synapses. **A and B** Experimental configuration, with the stimulating electrode (yellow) in the internal capsule, and recording electrode in the LA (**A**), or with an AAV expressing ChR2 and mCherry under the CaMKII promoter infused into the medial geniculate nucleus of the thalamus (MGM); blue light was applied (5 ms pulse, with 15 s interval) to their terminals in the LA (**B**). **C** ADX71743 increases evoked EPSC amplitude; traces at the top illustrate evoked EPSCs before, during, and following ADX71743 application (*n* = 10, 500 nM). **D** Quantification of peak normalized evoked EPSC amplitudes (one tailed paired Student’s *t* test, predrug vs ADX71743, t = 5.79¸***p < 0.0003, *n* = 10); grey lines and right-hand Y-axis denote absolute EPSC amplitudes. Traces at the top are representative examples. **E** Decreased paired-pulse ratio following ADX71743 application (500 nM) due to increase of the first peak of the pair (one tailed paired Student’s *t* test, predrug vs ADX71743, t = 4.12, **p = 0.0026, *n* = 10). **F** ADX71743 application (6 min, 500 nM) increases blue light evoked EPSC amplitude (*n* = 5). **G** Qua*n*tification of peak normalized evoked EPSC amplitudes; grey lines and right-hand Y-axis denote absolute EPSC amplitudes (paired Student’s *t* test, predrug vs ADX71743, t = 2.78, *p = 0.0499, *n* = 5). **H** ADX71743 (500 nM) reduced paired-pulse ratio of optically-evoked EPSCs in the LA cells shown in F and G (paired Student’s *t* test, predrug vs ADX71743, t = 5.13, *p < 0.0144, *n* = 4).

The sensory thalamus is an important relay station that conveys sensory inputs to the LA via the internal capsule [59, 60]. Because thalamic neurons express mGlu7 [58], and thalamus-to-LA synapses exhibit plasticity, store fear memories and their plasticity has been implicated in reconsolidation [61–63], we hypothesized that these synapses represent likely targets of ADX71743. We examined its effect, in rat brain slices, on the amplitude of EPSCs that were evoked by electrical stimulation of the internal capsule (Fig. 4A), or by optical stimulation of descending fibers from the medial geniculate thalamic nucleus (MGM) in the LA (Fig. 4B, Supplementary Fig S5; see Methods). MGM neurons relay specifically auditory sensory information to a subset of LA principal neurons and potentiation of MGM-to-LA synapses is important for auditory fear learning and memory [64, 65]. The evoked EPSCs (eEPSCs) were mediated by AMPA/kainate receptors since they could be blocked by 10 µM of CNQX (Supplementary Fig. S5A). Amplitude scaled linearly with stimulus intensity over the stimulation range tested (Supplementary Fig. S5A). For further testing an anticipated stimulatory effect of ADX71743 on eEPSC amplitude, we kept the stimulus intensity low (3 mA or 3 mW) to avoid a ceiling effect.

ADX71743 (500 nM, 6 min) increased eEPSC amplitude (Fig. 4C, D), peaking 2.5 min after ADX71743 application had commenced and returned to predrug values shortly after ADX71743 application had ended. The paired-pulse ratio of two electric stimuli at 40 ms interval caused a facilitation that significantly and reversibly decreased selectively in the same cells under ADX71743 (Fig. 4E), consistent with a presynaptic synaptic action of the compound. The results with electrical stimulation were recapitulated following optogenetic stimulation of MGM fibers in the LA, although it should be noted that, since our viral injection site appears to extend in part to posterior thalamic nuclei (Fig. S5), some opto-tagged fibers may have descended from posterior nuclei that convey nociceptive information. ADX71743 increased optogenetically-evoked EPSC amplitude (Fig. 4F, G), peaking 2 min after the onset of ADX71743 incubation and declining to predrug values following application. In these cells, paired-pulse ratio decreased from 1.3 ± 0.1 during baseline to 1.0 ± 0.1 during ADX71743 treatment (Fig. 4H), again consistent with a presynaptic mechanism of action. Taken together, it appears that, under these conditions of low stimulus intensity, negative allosteric modulation of mGlu7 disinhibits glutamate release from thalamic nerve terminals onto LA pyramidal neurons.

### ADX71743 prevents the induction of synaptic potentiation at the thalamus-to-LA synapse

**Fig. 5:**
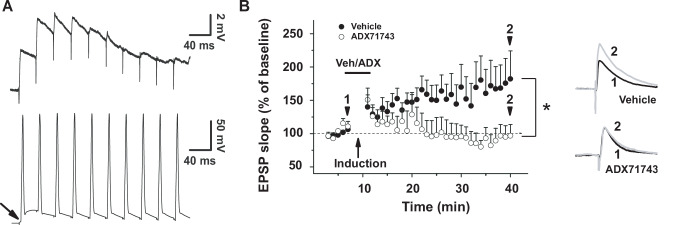
ADX71743 prevents LTP at thalamus-to-LA pyramidal neuron synapses. **A** LTP induction protocol consisting of 15 trains of stimuli, each containing 10 presynaptic electric stimulations at 30 Hz in the internal capsule that each were followed by a 5-ms postsynaptic depolarizing current of 1 – 1.5 nA applied 10 ms after the EPSP onset, *i.e*. at the time of EPSP peak amplitude. Stimulus trains were given at intervals of 10 s. Top, EPSPs (arrow, EPSP resulting from internal capsule stimulation), bottom action potentials. **B** LTP in slices treated with vehicle solution (0.005% DMSO in ACSF) or ADX71743 (500 nM); black and grey sample traces at the right indicate before and after LTP induction, respectively, denoted by corresponding numbers. LTP was completely prevented by ADX71743. One-tailed Student’s t-test, t = 2.061, *p = 0.0257, *n*(vehicle)=15, *n*(ADX71743) = 9.

Whereas the increased release of glutamate following ADX71743 incubation likely reflects modulation of constitutive, glutamate-independent mGlu7 activity, fear learning, memory and reconsolidation may rather rely on endogenous changes in glutamate-dependent synaptic transmission and ensuing plasticity. Therefore, we assessed whether ADX71743 can affect the induction of long-term potentiation (LTP) of thalamus-to-LA synapses [57] induced through a protocol in which endogenous released glutamate is paired with postsynaptic activation (see Methods and Fig. 5A). This protocol consisted of the pairing of EPSPs, evoked by 15 presynaptic trains of stimuli at 30 Hz in the internal capsule, with action potentials, evoked around the peak of the EPSPs, by depolarizing currents injected in the postsynaptic neuron 10 ms after EPSP onset [57]. Inhibitory currents were blocked by picrotoxin in the bath (50 µM).

In the absence of ADX71743, LTP was effectively induced, increasing the slope of the electrically evoked EPSP to 173 ± 37.2% of baseline (taken 30 min after induction, Fig. 5B). Bath-application of ADX71743 (500 nM) during the LTP induction protocol prevented LTP (Fig. 5B), demonstrating that presynaptic mGlu7 activation during high frequency stimulation is necessary for the induction of LTP.

### Negative allosteric modulation of mGlu7 promotes spontaneous presynaptic glutamate release in human brain tissue

To translate our findings to humans, we tested ADX71743 directly in human brain tissue that was obtained surgically from drug-resistant epileptic patients. Brain tissue was from the temporal neocortex and part of the basolateral amygdala that was removed prior to amygdalo-hippocampectomy as part of a standard anterior temporal lobectomy.

**Fig. 6:**
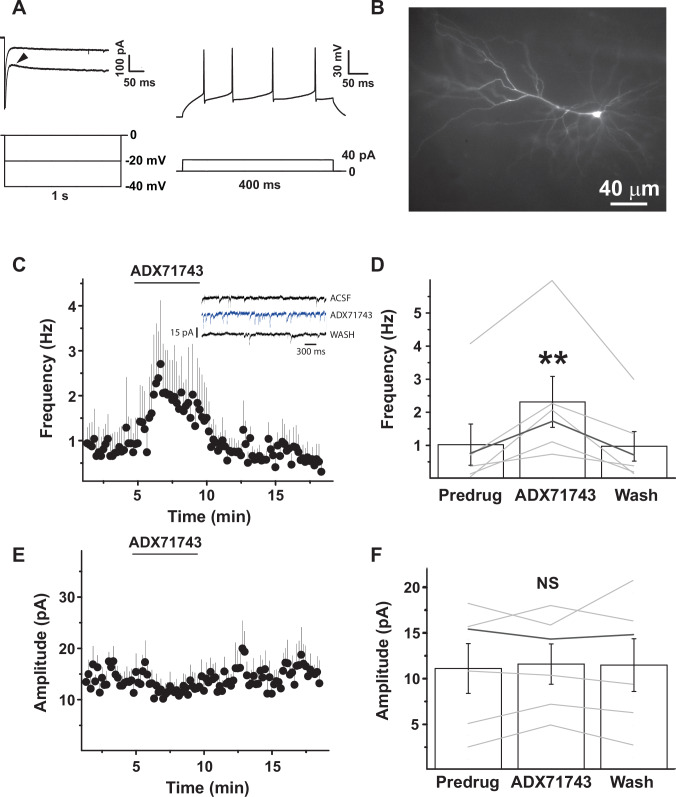
ADX71743 (500 nM) increases spontaneous EPSC frequency, but not amplitude, in human neurons. **A** Ih current (arrowhead) following 20 and 40 mV hyperpolarizations (left panel) and action potential frequency accommodation following injection of 40 pA current (right panel). **B** Biocytin staining after patch-clamping showing a human cortical pyramidal neuron with distinct triangular shape. **C** ADX71743 increases spontaneous EPSC frequency in five human cortical pyramidal neurons, and one LA neuron. Inset: representative EPSC recordings from a cortical neuron. **D** Mean effect of ADX71743 on spontaneous EPSC frequency, as assessed by peak frequencies for each cell, with the LA neuron shown in dark grey. Paired Student’s t-test, baseline vs ADX71743 application, t = 5.531, df = 5, *p* = 0.0013, n = 6; five cortical neurons and one amygdala neuron. **E** ADX71743 does not alter spontaneous EPSC amplitude in the six responding cells shown in C. **F** Quantification of effects of ADX71743 on spontaneous EPSC amplitude, taken when maximum frequency was reached in the cells shown in E. Paired Student’s *t* test, predug vs ADX71743, baseline vs ADX71743 application, t = 0.49, df=5, *p* > 0.05, same cells as in C and D.

The human cortical and amygdala neurons we recorded showed an Ih current following the injection of a hyperpolarizing current (Fig. 6A), were regular spiking neurons, and had a pyramidally shaped soma (Fig. 6B). The neurons that responded to bath-application of ADX71743 (see below) had a resting membrane potential of −67 ± 4 mV, a membrane resistance of 308 ± 101 MΩ, and a capacitance of 170 ± 33 pF. Bath application of ADX71743 (500 nM) increased spontaneous (*i.e*. in the presence of 1 µM TTX to prevent action potential-induced glutamate release) EPSC frequency on average from 1.0 ± 0.6 Hz to 2.3 ± 0.8 Hz (Fig. 6C, D). The increase of EPSC frequency in the human cells was transient, peaking at 2 min following the onset of ADX71743 treatment, and returning to baseline values once ADX71743 application had ended (Fig. 6C). sEPSC amplitudes were small (11 ± 3 pA) and not influenced by ADX71743 at the moment of peak EPSC frequency (Fig. 6E). Thus, ADX71743 appears to act presynaptically in human cortical and amygdala neurons, by enhancing spontaneous glutamatergic synaptic transmission. As sEPSC frequency is low, this probably reflects augmented constitutive activity of mGlu7. Together, these results are strikingly similar to those obtained in the rat LA, further validating the potential of mGlu7 negative allosteric modulation for future studies on reconsolidation in humans.

## Discussion

Disruption of fear memory reconsolidation has been proposed as a potential strategy in psychopharmacotherapy to weaken or even eliminate fear memories after a traumatic event or during phobias [35, 36, 66]. We found that the highly selective negative allosteric modulator of mGlu7, ADX71743, effectively disrupts fear memory reconsolidation in rats, and regulates glutamatergic neurotransmission in both rat and, importantly, human brain tissue. These findings hold significant translational promise for mGlu7 in the treatment of anxiety and fear disorders.

Multiple behavioral observations in the rat model indicate that the substantial decrease in freezing levels three days post-ADX71743 treatment is caused by impaired fear memory reconsolidation. Thus, we found that the decrease did not occur if the CS was not given prior to the application of ADX71743. Furthermore, it was specific to the CS, as we found that a parallel fear memory trace created by the conditioning to another CS2, that was recalled without subsequent ADX71743 infusion, was not affected. In addition, the fear-reduction only manifested itself when ADX71743 was infused within 4, but not 24 h after fear memory recall. These findings are consistent with previous reconsolidation studies that had identified a time window of psychotherapeutic treatments within a maximum 6 h after CS recall [25, 67]. Additionally, ADX71743 also reduced fear reinstatement, suggesting that its effect is distinct from fear extinction – the process underlying extinction-based therapies for PTSD and phobias. These therapies generate a safety memory trace while leaving the original fear memory trace intact [28, 68], allowing fear to resurface later through spontaneous recovery or reinstatement. In contrast, our findings indicate that ADX71743 acts on the reconsolidation process by keeping the fear memory in a destabilized state after recall. This can lead to a weakening – or even erasure - of the original memory trace. Thus, negative allosteric modulators of mGlu7 emerge as promising candidates for a novel approach to treating anxiety- and fear-related disorders.

The mechanism by which ADX71743 disrupts fear memory reconsolidation likely differs from that employed by the β-adrenergic receptor antagonist propranolol. Beta-adrenergic receptors in the LA recruit Ca^2+^-permeable AMPA receptors during cued fear conditioning [69], and, as activity of these receptors, newly inserted by retrieval, is required for reconsolidation [70–72], it is possible that propranolol acts on AMPA receptor exchanges to disrupt fear memory reconsolidation. β-adrenergic receptor activation can also permit induction of LTP at thalamus-to-LA synapses through inhibition of GABAergic interneurons [72], presenting another mechanism through which propranolol could impair reconsolidation. Unlike β-adrenergic receptors, mGlu7 is expressed presynaptically, where it can either inhibit glutamate release during low frequency stimulation through its initially proposed autoinhibitory action [19], or promote glutamate release during high frequency stimulation by strongly facilitating synaptic transmission [21]. Our electrophysiological recordings suggest that both mechanisms are at play in the LA. First, ADX71743 increased spontaneous EPSC (sEPSC) frequency and evoked EPSC (eEPSC) amplitude elicited by low stimulus frequency, which are both consistent with disinhibition of G_i/o_-coupled pathways. The reduced paired-pulse ratio in the presence of ADX7143 confirmed the presynaptic expression of the effects on glutamatergic synaptic transmission. Second, the complete block by ADX71743 of LTP induction by high frequency stimulation is consistent with the role of mGlu7 for inducing facilitation of glutamate release during high-frequency stimulation. As the block of LTP was observed in the presence of picrotoxin, it seems that, unlike propranolol, ADX71743 acts on synapses involving postsynaptic glutamatergic and not GABAergic signaling. A role of mGlu7 in synaptic plasticity has also been demonstrated pharmacologically with the orthosteric-like mGlu7 antagonist XAP044 in the LA [12], as well as with ADX71743 and the group III mGlu positive allosteric modulator VU0422288 in the hippocampus [73, 74]. Thus, our findings point to a presynaptic component involved in reconsolidation, in addition to the previously described postsynaptic component that relies of AMPA receptor exchange and protein synthesis [70, 75]. The question of how these pre- and postsynaptic components work in concert in synaptic processes underlying reconsolidation remains to be answered, but one could speculate that binding of presynaptic mGlu7 to postsynaptic Elfn2, which is, unlike Elfn1, probably expressed in glutamatergic neurons [23], integrates the regulation of presynaptic glutamate release with postsynaptic responses.

Our findings hold further translational value as we could extend the effects of ADX71743 on sEPSC frequency and amplitude from rodent to human neurons. This shows that functional mGlu7 is present in the human brain, exerting effects through a similar presynaptic mechanism as in the rodent brain. This further substantiates negative allosteric modulation of mGlu7 as a potential strategy to treat fear- and stress-related disorders in human patients. However, one limitation of the current study concerns the limited testing in healthy human, especially amygdala, brain tissue. We made use of brain tissue from temporal lobe epileptic patients who were resistant to pharmacological treatment. The amygdala is typically part of the epileptic focus and sclerotic as a result. Our electrophysiological recordings did not reveal any epilepsy-like activity in the human brain slices and yielded very similar results on spontaneous EPSC frequency as for the rat amygdala tissue. Moreover, there were no signs of epilepsy in the behavioral experiments on rats. Thus, even though mGlu7 loss of function mutations may underlie some forms of epilepsy in humans [75, 76], the brain tissue taken from the epileptic patients appears suitable for the study of effects of ADX71743 on glutamatergic transmission. The prevention of LTP by ADX71743 could perhaps be considered as a protection against epilepsy, as the heightened glutamatergic signaling following synaptic potentiation would not occur. However, dedicated early preclinical and clinical safety studies are warranted to demonstrate there is no increase in epilepsy risk with negative allosteric modulation of mGlu7 receptors.

Taken together, our behavioral and electrophysiological data indicate that negative allosteric modulation of mGlu7 may weaken fear-conditioned memories, by acting on glutamatergic signaling potentially underlying fear memory reconsolidation, at least in thalamus-to-amygdala synapses. It remains possible that inputs from other, cortical structures are involved, as inputs from somatosensory cortex, for example, express high levels of mGlu7 [14]. This would deserve further investigation considering postsynaptic changes in these have been associated with reconsolidation [77]. At the same time, inputs from the medial prefrontal cortex, involved in fear learning, memory and extinction, seem to be devoid of mGlu7 [15]. The specific targeting of activated glutamatergic synapses following recall may make negative allosteric modulators of mGlu7 ideal compounds to apply following fear memory recall during psychiatric interventions in the future, when patients relive the traumatic event, or are exposed to a frightening situation to recall phobia. Additional preclinical testing in more targeted animal models such as those for sustained fear [78] or PTSD [79], in older animals, in females (that have higher rates of PTSD [80]) or with stronger or more temporally distant stressors [81–83] is necessary for further validation of this concept. Our observation that ADX71743 exerts similar presynaptic effects in human brain tissue as in the rat LA suggests that negative allosteric modulation of mGlu7 holds translational promise as a target for future treatment of anxiety and fear disorders in which memory is an important feature.

## Supplementary information

S1

S2

S3

S4

S5

Supplementary Legends
