# Supplementary material for: Negative allosteric modulation of mGlu7 disrupts fear memory reconsolidation and glutamatergic signaling in rat and human brain tissue
Source: Mol Psychiatry. 2025 Dec 23;31(2):976–86. doi: 10.1038/s41380-025-03202-x (PMC12815652; doi:10.1038/s41380-025-03202-x)
Supplement: Supplementary file 6 — Supplementary Legends [file 41380_2025_3202_MOESM6_ESM.docx]

**Fig. S1. Injection sites and freezing responses for injections outside the LA. A** Localization of cannulas for both vehicle- (black dots) and ADX71743- (white dots) infused rats. Grey triangles and squares, ADX71743 infusions outside the LA. **B** Infusions outside the LA after recall on Day 2 did not reduce freezing in the retention test on Day 5.

**Fig. S2. ADX71743-reduced freezing in the retention test reflects disruption of reconsolidation rather than general fear reduction or impaired recall.** **A** Experimental paradigm. **B** ADX71743 infusion 10 min after the 5 kHz tone recall diminished freezing in the retention test, whereas vehicle infusion after the 15 kHz tone recall in the same animals did not, indicating a selective effect of ADX71743 on reconsolidation rather than a general reduction of fear or fear recall impairment. ADX71743 and vehicle were infused on different days per rat (day 2 or day 3 in a different context; see Methods). There were no differences between freezing following recall on the two days, indicating that a recall to a tone followed by ADX71743 or vehicle treatment on one day did not affect freezing 24h later (unpaired two-sided t-test, t=0.7892, df=16, *p*=0.4415). We have pooled the data for vehicle- and ADX71743-treated rats for analysis and graphic representation, and show freezing during recall across the two days in C. All animals were subjected to the retention test on day 5. A two-way ANOVA showed no interaction between the tone and the five CS presentations during the fear retention test on Day 5 (F(4,64)=0.595, *p*=0.6675) nor on CS (F(4,64)=1.65, *p*=0.1726). There was a significant effect of tone (F(1,16)=14.60, *p*=0.0015, Bonferroni-corrected for multiple comparisons), demonstrating that the disruption of fear memory reconsolidation by ADX71743 is specific to the stimulus for which it is applied, and not a generalized reduction of fear or impaired fear memory recall. ns, not significant, *p*=0.2092 (CS1), **p*=0.0481 (CS2), 0.0121 (CS3) or 0.0310 (CS5), ***p*=0.0026 (CS4). A pairwise comparison of freezing in the retention test averaged over the five CS presentations of both tones revealed that all rats but one freeze lower to the 5 kHz tone (ADX71743) than two the 15 kHz tone (vehicle). One-tailed paired t-test, t=3; df=8, ***p*=0.0074 *n*(vehicle) and *n*(ADX71743)=9. Colors correspond to those in C. **C** Detailed representation of freezing during recall on Days 2 and 3. Averaged freezing during habituation and to one of the tones during recall on day 2 and day 3 (left panel), or freezing levels to each tone for each individual animal during the two days (right panel). Symbols represent the tone (5 or 15 kHz), and colors represent the order of tone presentation (blue: 5 kHz on day 2 and 15 kHz on day 3; red: 15 kHz on day 2 and 5 kHz on day 3). For the right panel, ADX71743 (ADX) and vehicle (Veh) infusions after each tone presentation are indicated. Legend applies to both panels. There were no statistically significant differences within and between the two days of recall (5 kHz vs 15 kHz on day 2, paired two-tailed test, t=0.6044, df=4, *p*=0.5782; 5 kHz vs 15 kHz on day 3, t=0.8596, df=3, *p*=0.4532; 5 kHz on day 2 vs 5 kHz on day 3, unpaired two-tailed t-test, t=1.199, df=7, *p*=0.2696; 15 kHz on day 2 vs 15 kHz on day 3, unpaired two-tailed t-test, t=1.572, df=7, *p*=0.16; all 5 kHz vs 15 kHz tones, paired two-tailed t-test, t=0.2879, df=8, *p*=0.7807). **D** Injection sites in the dorsolateral part of the LA. Red dots indicate a cannula implantation outside the LA, corresponding to arrow in B.

**Fig. S3. Disruption of fear memory reconsolidation following ADX71743 injection 4h after fear memory recall.** ADX71743 infused in the LA bilaterally 4h after fear retrieval suppressed freezing during the fear memory retention test three days later. There was no interaction between tone vs habituation and the five CS presentations during the fear retention test on Day 5 (Two-way ANOVA, F(5,50)=2.133, p=0.0767). ADX71743 infusion 4h after fear memory recall on Day 2 reduced freezing on Day 5 when compared with vehicle-injected animals in the fear retention test (Two-way ANOVA with Bonferroni correction, F(1,10)=37.10, *p*=0.0001), indicating that fear memory reconsolidation can be disrupted by ADX71743 four hours after fear memory recall. **p*=0.0398 (CS1) or 0.0124 (CS4), ***p*=0.0044 (CS2), 0.0066 (CS3) or 0.0024 (CS5). *n*(vehicle) and *n*(ADX71743)=6. The two dots on the right indicate averaged freezing over five CS presentations of two animals used in the same experiment that had received ADX71743 10 min after recall on day 2.

**Fig. S4. The disruption of fear memory reconsolidation by subcutaneous injected ADX71743 is quantitatively similar to that induced by intraperitoneally injected propranolol.** ADX71743 injections on Day 2 after fear memory recall significantly reduces freezing to CS1 and CS2 in the fear retention test, whereas the effect of propranolol (10 mg/kg bodyweight, in 1 ml of saline) is significant only to CS2. Two-way repeated measures ANOVA, F(2,32)=4.124; *p*=0.0255, *p*<0.05 for ADX71743 vs vehicle; #*p*<0.05 for propranolol vs vehicle. Vehicle is pooled for subcutaneous and intraperitoneal injections. *n*(vehicle)=11, *n*(ADX71743)=12, *n*(propranolol)=12.

**Fig. S5. Characterization of responses to electric and optogenetic stimuli. A** Electrically-evoked EPSCs are mediated by AMPA glutamate receptors (blocked by CNQX), and increase linearly with stimulus intensity. **B** EPSC amplitude as a function of blue light stimulus intensity. **C** Representative example of mCherry expression in MGM neurons four weeks after AAV infusion. **D** Blue light stimulation in the LA induced EPSPs, occasionally triggering an action potential (top), and always facilitating EPSCs (bottom) in LA pyramidal neurons. A representative example is shown.
